# LMAN2 Promotes Breast Cancer Tumorigenesis and Drug Resistance by Interacting With MAPK9 via Activation of the MAPK Pathway

**DOI:** 10.1002/cam4.70448

**Published:** 2024-12-02

**Authors:** Chen Feng, Pingping Li, Pengtao Liu, Bo Wang, Juan Li, Peijun Liu

**Affiliations:** ^1^ Center for Translational Medicine The First Affiliated Hospital of Xi'an Jiaotong University Xi'an China; ^2^ Department of Oncology Shaanxi Provincial Corps Hospital Xi'an China

**Keywords:** breast cancer, LMAN2, MAPK pathway, MAPK9, tumorigenesis

## Abstract

**Introduction:**

Breast cancer (BC) is the most prevalent malignancy among women worldwide. Lectin, mannose‐binding 2 (LMAN2) is a cargo receptor engaged in the transport and sorting of glycoproteins. Despite its ubiquity, the function and underlying mechanisms of LMAN2 in BC continue to elude understanding.

**Methods:**

Multiple databases were employed to examine the expression of LMAN2 in breast cancer. Immunohistochemistry(IHC), qRT‐PCR, and Western blot were performed to quantify LMAN2 expression in BC cell lines and clinical samples. Heat map analysis and Kaplan–Meier analysis were used to analyze the correlation between LMAN2 and clinicopathological features. SiRNAs and overexpression plasmids were transfected into two BC cells to assess the effect of LMAN2 on malignant phenotypes. Coimmunoprecipitation and immunofluorescence were used to screen for potential interacting proteins. Additionally, tumor subcutaneous xenograft mode was constructed to explore tumor chemoresistance.

**Result:**

LMAN2 expression was significantly higher in BC compared to that in matched, adjacent normal tissues, and its higher expression level was correlated with worse patient prognosis. In vitro, we found that LMAN2 functions as an oncogene, promoting BC cell proliferation, cell cycle progression, invasion, and chemoresistance while preventing apoptosis. Coimmunoprecipitation and colocalization experiments confirmed the direct binding of LMAN2 to MAPK9 in BC cells. Our investigation of signaling pathways suggested that LMAN2 is involved in the regulation of the MAPK signaling pathway, utilizing this pathway to confer cisplatin resistance. Furthermore, knockdown of LMAN2 improves the sensitivity of drug‐resistant BC cells to cisplatin (DDP) in vivo.

**Conclusion:**

LMAN2 was a novel diagnostic and prognostic biomarker for BC that promotes chemoresistance via interaction with MAPK9 and activation of the MAPK pathway.

## 1 Introduction

The prevalence and mortality rate of breast cancer (BC), the foremost cancer impacting women's health globally, continue to increase in underdeveloped nations. According to 2020 global cancer statistics, BC is the most common malignant tumor in women, accounting for 11.7% of all cancer types [1]. Owing to its high heterogeneity at the molecular and cellular levels, BC displays potentially aggressive characteristics and complex biological features [2]. Early detection and precise treatment remain the most effective approaches to combatting cancer, both of which rely on a profound understanding of the genetics and origin of BC. The complex pathogenesis of BC originates from changes in various genes, hormone receptors, and epigenetics. Therefore, identifying effective diagnostic and prognostic markers is critical for improving the early diagnosis rate and prolonging survival.

Lectin, mannose‐binding 2 (LMAN2), a protein‐coding gene, is responsible for encoding a type I transmembrane lectin [3]. It is located mainly on the plasma membranes of various cytoplasmic organelles, such as the endoplasmic reticulum (ER) and Golgi apparatus, and acts as a cargo receptor in the transport and sorting of glycoproteins. The biological functions of LMAN2 in cells are related to the early secretory pathway, protein metabolism, and Golgi transport dynamics [4]. Given that most research focusing on LMAN2 has concentrated on its protein transport functionality, its role in malignancy remains unknown. In ovarian cancer, patients with high expression of LMAN2 show better overall survival benefit after intraperitoneal (IP) chemotherapy than adjuvant intravenous (IV) platinum‐taxane chemotherapy [5]. It has also been shown to be highly expressed in gastric cancer [6] and brain metastatic BC [7]. Moreover, bioinformatics analysis revealed that LMAN2 may be closely related to BC stemness, metastasis, differentiation, and the DNA damage repair pathway in HER2+ BC [8]. However, the roles and molecular mechanisms of LMAN2 in BC initiation and progression remain to be clarified.

Mitogen‐activated protein kinases (MAPKs) are a conserved family of serine/threonine protein kinases that play key roles in transducing extracellular signals into a wide variety of cellular responses [9]. MAPKs consist of three known subtypes, including ERK1/2, JNK1/2, and p38. It has been reported that the MAPK cascade mediates several basic cellular processes, including cell growth, adhesion, survival, and differentiation, across malignancies [10]. Previous studies revealed that PLD4 can activate the MAPK signaling pathway after binding to LMAN2, thereby inducing kidney fibrosis [11]. However, there have been no reports on the function of LMAN2 in the regulation of MAPK signaling in BC.

In this study, we revealed that LMAN2 expression was significantly greater in BC tissues than in adjacent tissues and was closely associated with poorer BC patient outcomes. Our in vitro experiments confirmed that LMAN2 expression enhances the malignant phenotype and chemotherapy resistance of BC cells. Moreover, LMAN2 facilitates chemotherapy resistance in BC by activating the MAPK signaling pathway. These findings offer insight into the possible mechanisms underlying LMAN2‐induced chemotherapy resistance in BC and can aid in identifying potential therapeutic targets for combating BC.

## 2 Methods and Materials

### 2.1 Database Analysis

Bioinformatic data were obtained from multiple public databases. The normalized RNA‐seq data and clinical information of patients with BC were downloaded from the TCGA website (https://cancergenome.nih.gov/) [12]. Tumor immune estimation resource 2.0 (TIMER2.0) database (http://timer.cistrome.org/) was used to analyze the mRNA expression levels of LMAN2 between tumor samples and adjacent normal tissues across all TCGA tumors in “Gene_DE” module [13, 14]. Kaplan–Meier plotter (www.kmplot.com) [15] were then used to analyze the overall survival (OS) in TCGA‐BRCA and Tang_2018 database. The detailed steps were as follows: mRNA/other➔ Start KM Plotter for breast cancer/Start KM Plotter for breast protein➔ Gene ➔ symbol ➔ LMAN2 ➔ Survival ➔ OS ➔ Draw Kaplan–Meier plot. METABRIC breast cancer data and survival information were downloaded from cBioPortal (https://www.cbioportal.org/) [16]. The detailed steps for METABRIC database analysis were as follows: click “cBioPortal” website ➔ click “Data SET” ➔ search “METABRIC” ➔ download “Data_mRNA_median_Zscores.txt” and “data_clinical_sample.txt” SCAN‐B BC data and survival information were downloaded from the NCBI Gene Expression Omnibus (https://www.ncbi.nlm.nih.gov/geo/query/acc.cgi?acc=GSE60789) [17]. The protein expression matrix of the TCGA‐BRCA cohort was obtained from CPTAC (https://cptac‐data‐portal.georgetown.edu/datasets) [18]. The detailed steps for CPTAC database analysis were as follows: Analysis ➔ cProsite ➔ TumorTypes ➔ Breast cancer ➔ Dataset ➔ Relative Protein Abundance ➔ Analysis ➔Tumor vs. Normal Tissue ➔ Gene ➔ LMAN2 ➔Tumorview. Representative immunohistochemistry (IHC) images of LMAN2 staining were retrieved from the Human Protein Atlas (HPA) database (http://www.proteinatlas.org) [19]. The detailed steps for HPA database analysis were as follows: enter “LMAN2” ➔ click “search” ➔ click “Pathology”/“Tissues” ➔ click “Cancer”/“Tissues” ➔ choose “BREAST CANCER”/“BREAST” ➔ obtain representative IHC pictures. BC single‐cell sequencing datasets were obtained from CancerSEA (http://biocc.hrbmu.edu.cn/CancerSEA/) [20]. The detailed steps for CancerSEA database analysis were as follows: Search ➔ Input a gene ➔ enter “LMAN2” ➔ click “search” ➔ obtain “correlation plot.” Correlation analysis of LMAN2 and MAPK pathway‐related proteins was performed via the GEPIA database (http://gepia.cancer‐pku.cn) [21]. The detailed steps for GEPIA database analysis were as follows: Enter gene name ➔ LMAN2 ➔Correlation ➔ GeneA ➔ LMAN2 ➔ GeneB ➔ MAPK pathway‐related genes ➔ Correlation Coefficient ➔ Spearman ➔ TCGA tumor ➔ BRCA tumor ➔Add ➔ Plot.

### 2.2 Patients and Tissue Samples

Human tissue microarrays (TMAs, HBreD090CS01) of breast cancer tissues containing 45 pairs of BC tissues and nontumor adjacent tissues were obtained from Shanghai Biochip Company (Shanghai, China). The study was approved by the Ethics Committee of the Shanghai Outdo Biotech Company ethics committee.

### 2.3 Chemical Reagents

Primary antibodies against LMAN2 (abs152187), MAPK9 (abs115519), and IgG (abs20161) were purchased from Rom Absin Biochemical Company (Absin, Shanghai, China); primary antibodies from an EMT Antibody Sampler Kit (#9782) and GAPDH (#2118) were obtained from Cell Signaling Technology (Danvers, MA, USA); primary antibodies against P38 (66234‐1‐Ig) and p‐P38 (28796‐1‐AP) were obtained from Proteintech (Wuhan, China); and primary antibodies against MRP1 (ab260038), MDR1 (ab170904), JNK (ab208035), p‐JNK (ab47337), and p‐ERK (ab314200) were obtained from Abcam (Abcam, Cambridge, UK). A CCK‐8 detection kit (#C0037) and cell cycle and apoptosis analysis kits (#C1052) were purchased from Beyotime Biotechnology (Shanghai, China). Cisplatin (CAS No: 15663‐27‐1) was purchased from MedChemExpress (Monmouth Junction, NJ, USA). The p38MAPK agonist P79350 (50 mmol/L; Calbiochem, La Jolla, CA, USA) was used. Pierce Protein A/G Agarose (#20421) was obtained from Thermo Fisher Scientific (Waltham, USA).

### 2.4 Cell Lines and Transfection

The human BC cell lines BT‐474, MDA‐MB‐231, MCF‐7, T47D, and HCC1937 were purchased from ATCC. Human mammary epithelial MCF‐10A cells and DDP‐resistant MCF‐7 cells were obtained from the Shanghai Chinese Academy of Sciences Cell Bank (Shanghai, China). MCF‐10A cells were cultured in MEGM (Lonza, Walkersville, MD, USA). MCF‐7 cells were cultured in modified Eagle's medium (HyClone, Logan, UT, USA). BT‐474 and MDA‐MB‐231 cells were cultured in Dulbecco's modified Eagle's medium (DMEM, HyClone, Logan, UT, USA). T47D and HCC1937 cells were cultured in RPMI 1640 medium (HyClone, Logan, UT, USA). All the above‐listed media were supplemented with 10% fetal bovine serum (FBS, Gibco) and 1% penicillin–streptomycin (Gibco). To maintain the resistance phenotype, MCF‐7/DDP cells were cultured in the presence of 2 mM cisplatin. Small interfering targeting LMAN2 and negative control siRNA was obtained from Hanheng Biotechnology (Hanheng Biotechnology Co. Ltd., Shanghai, China). An LMAN2‐cDNA lentiviral vector and an empty lentiviral vector were purchased from Gene Pharma Company (Shanghai, China). The siRNAs sequence employed in this study was listed in Table 1. Supernatants containing lentiviruses were used to infect BC cell lines overnight. After 72 h of culture, successfully infected cells were further selected using 2.5 μg/mL puromycin for at least 1 week. After screening, the remaining cells were immediately frozen in liquid nitrogen and then stored at −80°C for subsequent experiments.

**TABLE 1:** The siRNA sequences for indicted genes.

LMAN2	Sense sequence	Antisense sequence
siRNA1	CGAGCGAACUGGAUCUUUAUU	UAAAGAUCCAGUUCGCUCGGU
siRNA2	CCAGGUGAGUGGUUCUCAAGA	UUGAGAACCACUCACCUGGUG

### 2.5 Quantitative Real‐Time PCR (qRT‐PCR)

Total RNA was extracted from two transfected BC cells using Trizol reagent (Beyotime, Shanghai, China) according to the manufacturer's instructions. RNA reverse transcription to cDNA was performed using TaqMan reverse transcription reagents(Takara Biotechnology). qRT‐PCR analysis was carried out with SYBR‐Green PCR master mix (Takara, Dalian, China). Finally, mRNA expression levels of LMAN2 were calculated using the 2^−ΔΔCT^ method, and GAPDH served as the internal control for RNA normalization. The detailed primer sequences of LAMN2 and GAPDH are listed in Table 2.

**TABLE 2:** Human primer sequences used for qRT‐PCR.

Primers	Forward (5′–3′)	Reverse (5′–3′)
LMAN2	ACTGGTGACCTGTCTGACAAT	ACACTGGGCTCAATCTTGGT
GAPDH	TGATGGGTGTGAACCACGAG	CCCTTCCACGATGCCAAAGT

### 2.6 CCK‐8 and Cell Viability Assay

BC cells (3000/well) from different treatment groups were incubated in 96‐well plates overnight. At each time point (0, 24, 48, 72, and 96 h), 10 μL of CCK‐8 reagent was added to each well, and the plates were incubated at 37°C for 1 h. Afterward, the optical density (OD) of each sample was detected by a microplate reader (Bio‐Rad Laboratories, Hercules, CA, USA) at a wavelength of 492 nm. The cell viability of two transfected BC cells and their parental cells was measured by sulfated carboxyl‐terminal CCK octapeptide (CCK‐8) assay. In brief, if all cells were attached, which could be separately treated with Cisplatin (DDP). DDP was soluble in N,N‐dimethylformamide (DMF) (2 mg/mL) then in a complete cell culture medium to a final concentration of 20,40,60 80, and 100 μM. After 48 h of treatment, the cells were incubated with 10% CCK8 solution for 3 h at 37°C. The optical density of the survival cells was determined at 492 nm in a micro‐plate reader. Relative cell viability was calculated by the following formula: Cell viability % = [OD 492 (treated) − OD 492 (blank)]/[OD 492 (control) − OD 492 (blank)] × 100%.

### 2.7 Colony Formation Assay

BC cells (300/well) were seeded into six‐well plates and cultured for 2 weeks. Then, the colonies were washed with PBS three times, fixed with 4% paraformaldehyde for 15 min, and stained with 1% crystal violet (V5265, Sigma–Aldrich, United States) for 5 min. Visible clones were manually counted.

### 2.8 Wound‐Healing Assay

BC cells were seeded in 6‐well plates at a density of 2 × 10^6^ cells/well and cultured until 90% confluence. Then, the plates were uniformly scratched with a sterile 200‐μL micropipette tip and washed with PBS twice to remove the scratched cells. The mean width of each scratch was measured and calculated with ImageJ software (NIH; version 1.4).

### 2.9 Transwell Migration and Invasion Assays

The migration and invasion of BC cells were assessed with 24‐well Transwell chambers (8 μm pore size, Corning, NY, USA). For the migration assay, the upper chamber was filled with 200 μL of serum‐free medium containing 5 × 10^5^ cells, while 600 μL of complete medium containing 10% FBS was added to the bottom chamber. The Transwell invasion assay procedure was similar to that used for the migration assay, except that the membrane in the upper chamber was precoated with a layer of 0.5% Matrigel (Becton Dickinson, NJ, USA). After culturing for 24 h, the migratory or invasive cells were methanol‐fixed, stained with 1% crystal violet, photographed, and counted under a light microscope (×200).

### 2.10 Cell Cycle and Apoptosis Analysis

Transfected BC cells were harvested by centrifugation with cold PBS and then fixed in ice‐cold 70% ethanol overnight at 4°C. Then, the cells were treated with RNase A (1 mg/mL) for 30 min at 37°C to degrade intracellular RNA, followed by DNA staining with propidium iodide (50 μg/mL) for 30 min on ice in the dark. A flow cytometer (Becton Dickinson, Canton, MA, USA) was used to detect the fluorescence signal at an excitation wavelength of 488 nm, and 10,000 cells were recorded for each sample. An apoptosis assay was performed using an apoptosis detection kit (Beyotime, #C1052) according to the manufacturer's instructions. The percentage distribution of apoptotic cells was determined by a BD FACSCalibur flow cytometer.

### 2.11 Immunofluorescence Staining

BC cells (1.0 × 10^5^ cells/well) were seeded on a sterile coverslip (24 × 24 mm) in a six‐well plate for 24 h. After being washed with ice‐cold PBS, the cells were fixed in 4% paraformaldehyde for more than 15 min at room temperature. Then, the sections were incubated with primary antibody overnight at 4°C. After incubation with secondary antibodies, the slides were counterstained with 4′,6‐diamidino‐2‐phenylindole to stain the cell nuclei. Fluorescence images were observed under a Leica fluorescence microscope (DM4000).

### 2.12 Western Blot and IP Assays

Total protein was extracted from BC cells using RIPA lysis buffer containing protease inhibitors and phosphorylase inhibitors. The supernatants were mixed with 5 × Western blot loading buffer and boiled for 5 min at 100°C. Then, the extracted total proteins were separated by SDS–PAGE (sodium dodecyl sulfate–polyacrylamide gel electrophoresis) and transferred to methanol‐activated PVDF membranes. Primary antibodies against LMAN2 (dilution 1:500), E‐cadherin (dilution 1:1000), N‐cadherin (dilution 1:1000), Snail1 (dilution 1:500), vimentin (dilution 1:1000), MRP1 (dilution 1:500), MDR1 (dilution 1:500), GAPDH (dilution 1:1000), MAPK9 (dilution 1:500), ERK (dilution 1:1000), p‐ERK (dilution 1:500), JNK (dilution 1:1000), p‐JNK (dilution 1:500), p‐ERK (dilution 1:500), P38 (dilution 1:1000), and p‐P38 (dilution 1:500) were diluted in accordance with the recommended protocols. Then, the PVDF membranes were incubated with these primary antibodies at 4°C overnight and then incubated with secondary antibodies at room temperature for 1 h. The membranes were washed thoroughly with TBST three times. Finally, the bands were visualized with an enhanced chemiluminescence system using a BeyoECL Plus kit (P0018, Beyotime). For the IP assay, the cells were lysed using weak‐potency NP‐40 lysis buffer (Beyotime) for 30 min on ice and then centrifuged at 15,000 rpm for 30 min at 4°C to collect the supernatants. The supernatant of each sample was incubated with anti‐LMAN2 at 4°C overnight. An anti‐IgG antibody without a primary antibody was used as a negative control. Subsequently, the lysate antibody mixture was incubated with prewashed protein A/G agarose and incubated at 4°C for 6 h. After washing with elution buffer, the immunoprecipitates were boiled and subjected to Western blot analysis.

### 2.13 In Vivo Tumorigenesis Assay

Four‐week‐old female BALB/c nude mice were purchased from the Kunming Institute of Zoology of the Chinese Academy of Science. The ethics committee of The First Affiliated Hospital of Xi'an Jiaotong University approved the experimental animal protocols, which were performed according to the National Institutes of Health (NIH) guidelines. Twelve mice were randomly divided into three distinct groups: the shControl group, shLAMN2 group, and shLAMN2+ P79350 group. Preprocessed MCF‐7/DDP cells (6 × 10^6^ cells/200 μL) were subcutaneously injected into nude mice. When the tumor volumes of the shControl group mice reached 50 mm^3^, the mice were intraperitoneally injected with DDP (5 mg/kg) every 3 days for 15 days. The tumor volume was measured every week using the formula 0.5 × length×width^2^. The mice were sacrificed on Day 30, and the weights of the xenograft tumors were recorded.

### 2.14 Statistical Analysis

Independent experiments were performed in triplicate and repeated at least three times. All quantitative data are expressed as the mean ± SEM, while qualitative variables are expressed as counts and percentages. SPSS 22.0 (SPSS, Chicago, IL, USA) and GraphPad Prism 9.5 (GraphPad Software, San Diego, CA, USA) were used for the statistical analyses. Comparisons of data between two groups were analyzed using Student's t‐test, and comparisons among more than two groups were analyzed using ANOVA. *p* < 0.05 was considered to indicate statistical significance. Gene set enrichment analysis (GSEA) was conducted using Java desktop software (http://software.broadinstitute.org/gsea/index.jsp) [22].

## 3 Results

### 3.1 LMAN2 is Upregulated in BC Tissues and is Associated With Poor Prognosis

**FIGURE 1:**
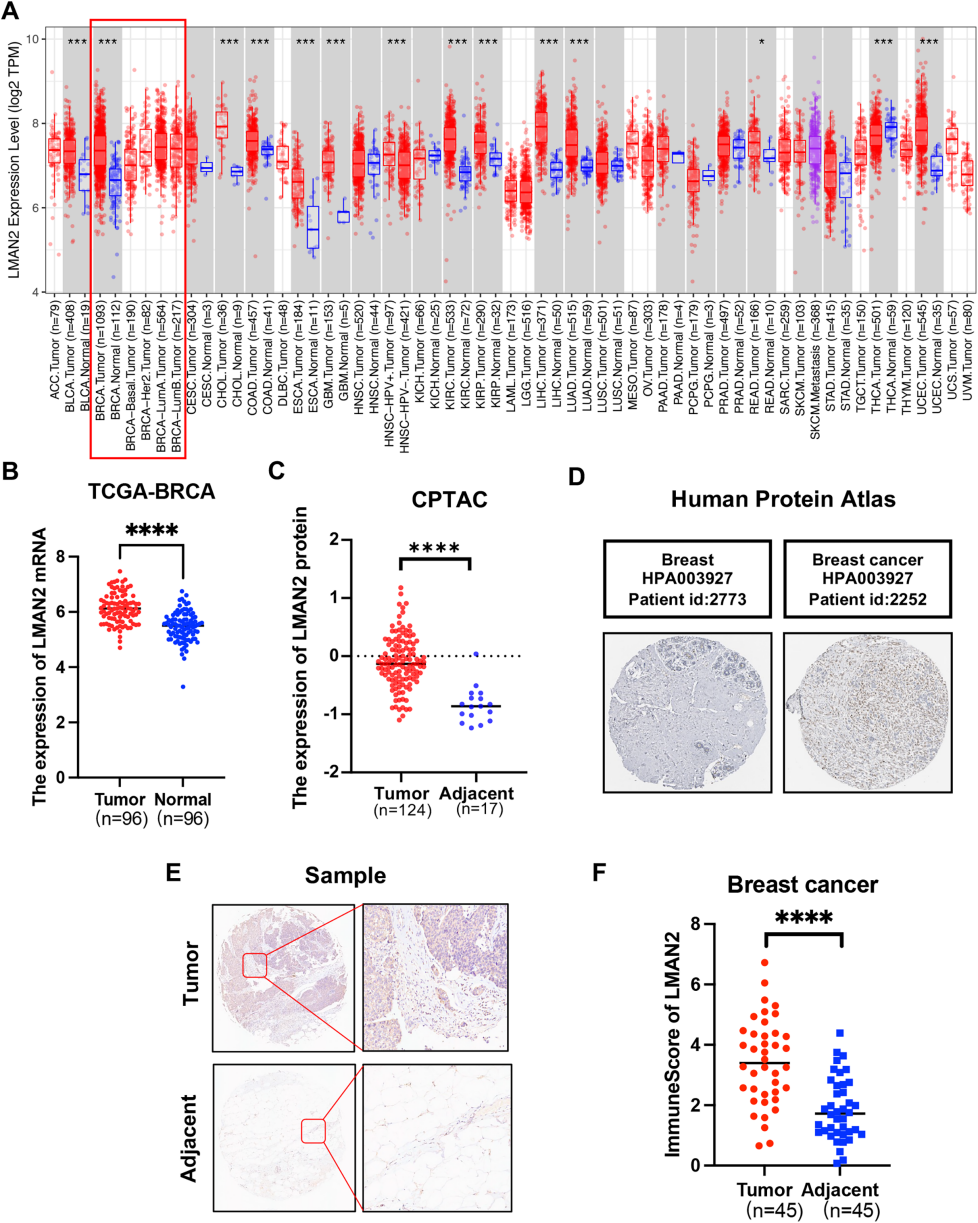
LMAN2 is upregulated in BC tissues. (A) Expression profiles of LMAN2 in multiple cancer tissues and corresponding adjacent normal tissues were analyzed via the TIMER database. (B) Comparative analysis of LMAN2 expression between BC tissues and paired paracancerous tissues from the TCGA‐BRCA database. (C) Quantitative analysis of LMAN2 protein expression using data from the CPTAC database. (D) IHC images of LMAN2 were obtained from the HPA database. (E)Representative images of IHC staining of LMAN2 in BC tissues and corresponding adjacent noncancerous tissues from TMA tissue sections (*n* = 45). (F) Statistical analysis of LMAN2 IHC staining scores in 45 cases of BC tissues. *p < 0.05, **p < 0.01, ***p < 0.001, ****p < 0.0001.

**FIGURE 2:**
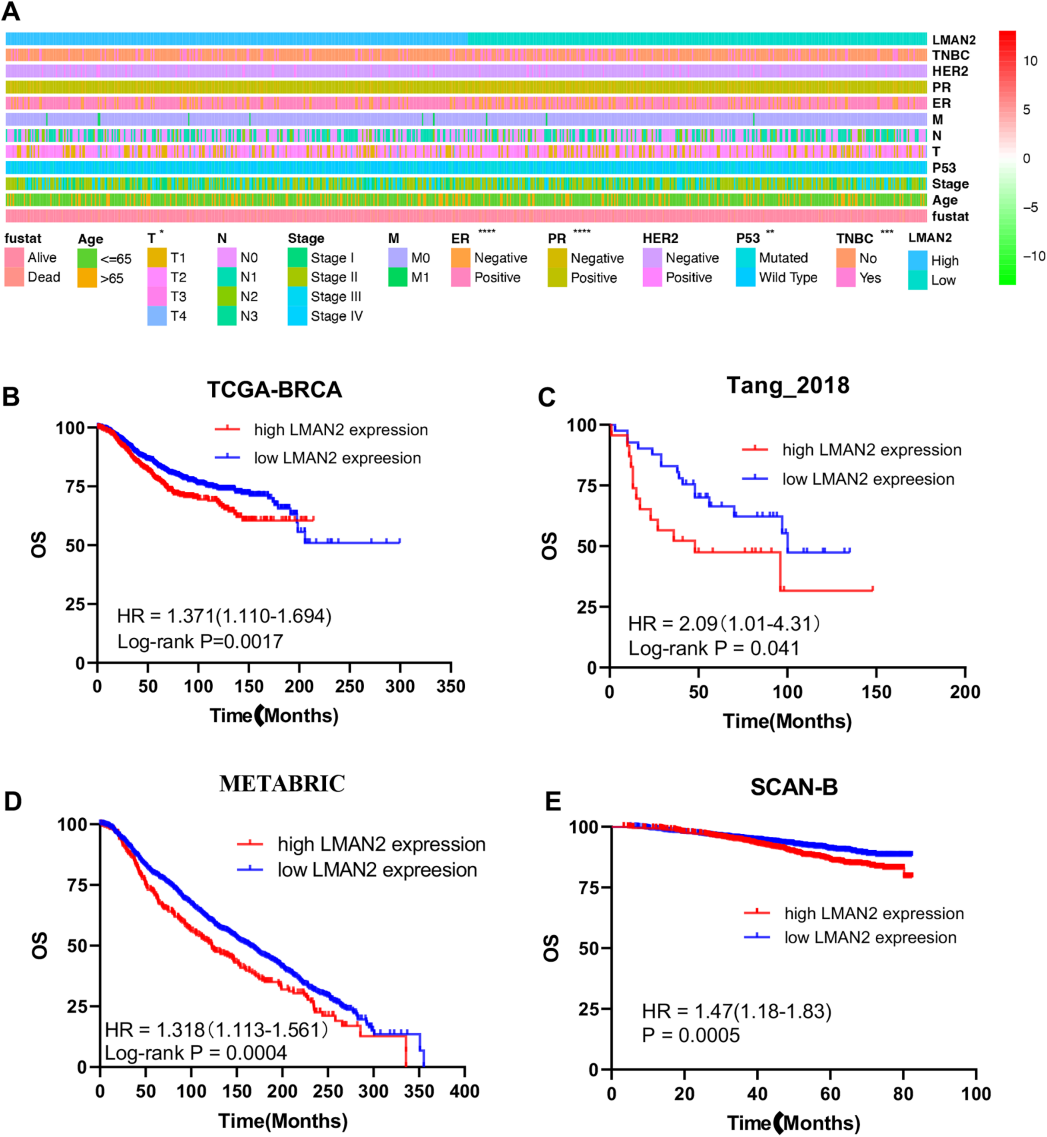
Relationship between clinical features and LMAN2 expression. (A) The strip chart shows the differences in the distribution of clinical features between the high‐ and low‐LMAN2 expression groups. (B–E) Kaplan–Meier analysis of the association between LMAN2 expression and overall survival in BC patients in the TCGA, Tang_2018, METABRIC, and SCAN‐B cohorts. The data are shown as the means ± SEMs of at least three experiments. *p < 0.05, **p < 0.01, ***p < 0.001, ****p < 0.0001.

We first used the TIMER database (https://cistrome.shinyapps.io/timer/) for pancancer analysis of LMAN2 expression across 33 types of cancer tissues in comparison with their corresponding normal tissues. The data revealed significantly elevated expression of LMAN2 in the majority of cancer tissue types compared to adjacent normal tissues (Figure 1A). LMAN2 mRNA expression was also significantly greater in BC tissues than in paired noncancerous tissues according to the TCGA‐BRCA microarray database (Figure 1B). Upregulated LMAN2 protein expression in BC tissues was also verified using the CPTAC database (Figure 1C). Similar LMAN2 expression imbalances were confirmed by immunohistochemistry (IHC) of samples from the Human Protein Atlas (HPA) database (https://www.proteinatlas.org/) (Figure 1D). Examination of breast tissues from the TMA confirmed higher levels of LMAN2 expression in BC tissues, as evidenced by IHC (Figure 1E,F). Subsequently, this study investigated the correlation between LMAN2 expression and various clinicopathological features in patients from the TCGA‐BRCA database. BC patients were categorized into high and low groups based on Cutoff Finder parameters, revealing a close correlation between high LMAN2 expression and T stage, ER status, PR status, TNBC status, and P53 mutation status (Figure 2A). Survival analysis further revealed high LMAN2 expression as an indicator of poor prognosis in BC patients from multiple databases, including the TCGA‐BRCA (Figure 2B), Tang_2018 (Figure 2C), METABRIC (Figure 2D), and SCAN‐B databases (Figure 2E). These observations suggest a possible role for LMAN2 as a potential diagnostic and prognostic biomarker for BC.

### 3.2 LMAN2 Promotes Cell Proliferation and Cell Cycle Progression and Reduces Apoptosis in BC Cells

**FIGURE 3:**
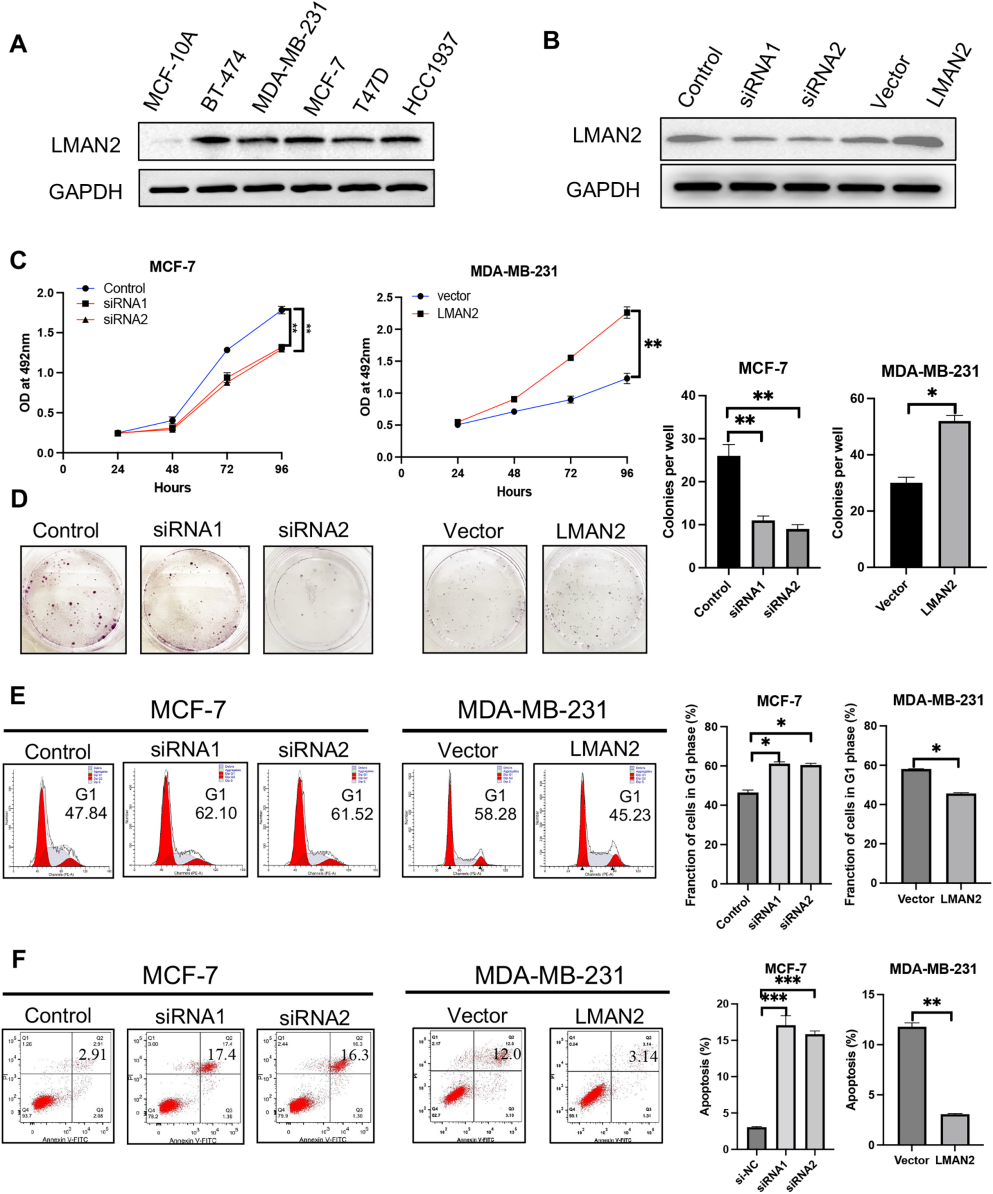
LMAN2 promotes cell growth. (A) Expression of LMAN2 in five BC cell lines and in the human mammary epithelial cell line MCF‐10A. (B) The transfection efficiencies of sh‐LMAN2 in MCF‐7 cells and pc‐LMAN2 in MDA‐MB‐231 cells were determined by Western blot. (C) A CCK‐8 assay was performed to detect the proliferation ability of the transfected BC cells. (D) A colony formation assay was performed to detect the colony formation abilities of the transfected BC cells. (E) Flow cytometry analysis of the cell cycle distribution of the transfected BC cells. (F) Flow cytometry analysis of cell apoptosis in the transfected BC cells. Quantitative analysis of cell proliferation, cell cycle progression, and apoptosis are shown in the right panel. The data are shown as the means ± SEMs of at least three experiments. *p < 0.05, **p < 0.01, ***p < 0.001.

To investigate the biological functions of LMAN2 in BC cells, we first determined the expression of LMAN2 in five BC cell lines and nonmalignant breast epithelial MCF‐10A cells. As expected, greater expression of LMAN2 was detected in breast cancer cells than in MCF‐10A cells (Figure 3A). Subsequently, expression of the LMAN2 gene was either knocked down or overexpressed in two BC cell lines (MCF‐7 and MDA‐MB‐231) via lentiviral technology. The expression of LMAN2 in the stable cell line was verified by qRT‐PCR and Western blot analysis (Supplemental Figure 1 and Figure 3B). We then explored the cell proliferation ability through a Cell Counting Kit‐8 (CCK‐8) assay and colony formation assays in the transfected cells. LMAN2 expression knockdown suppressed cell proliferation and colony formation in the MCF‐7 cell line, while LMAN2 overexpression had the opposite effect on the MDA‐MB‐231 cell line (Figure 3C,D). Flow cytometry analysis of the cell cycle revealed that LMAN2 knockdown arrested the cell cycle in the G1 phase, while LMAN2 overexpression facilitated the cell cycle transition from the G1 to the S phase (Figure 3E). Apoptosis analysis demonstrated that LMAN2 knockdown promoted apoptosis in MCF‐7 cells, while LMAN2 overexpression inhibited apoptosis in MDA‐MB‐231 cells (Figure 3F).

### 3.3 LMAN2 Promotes Cell Migration, Invasion, and EMT

**FIGURE 4:**
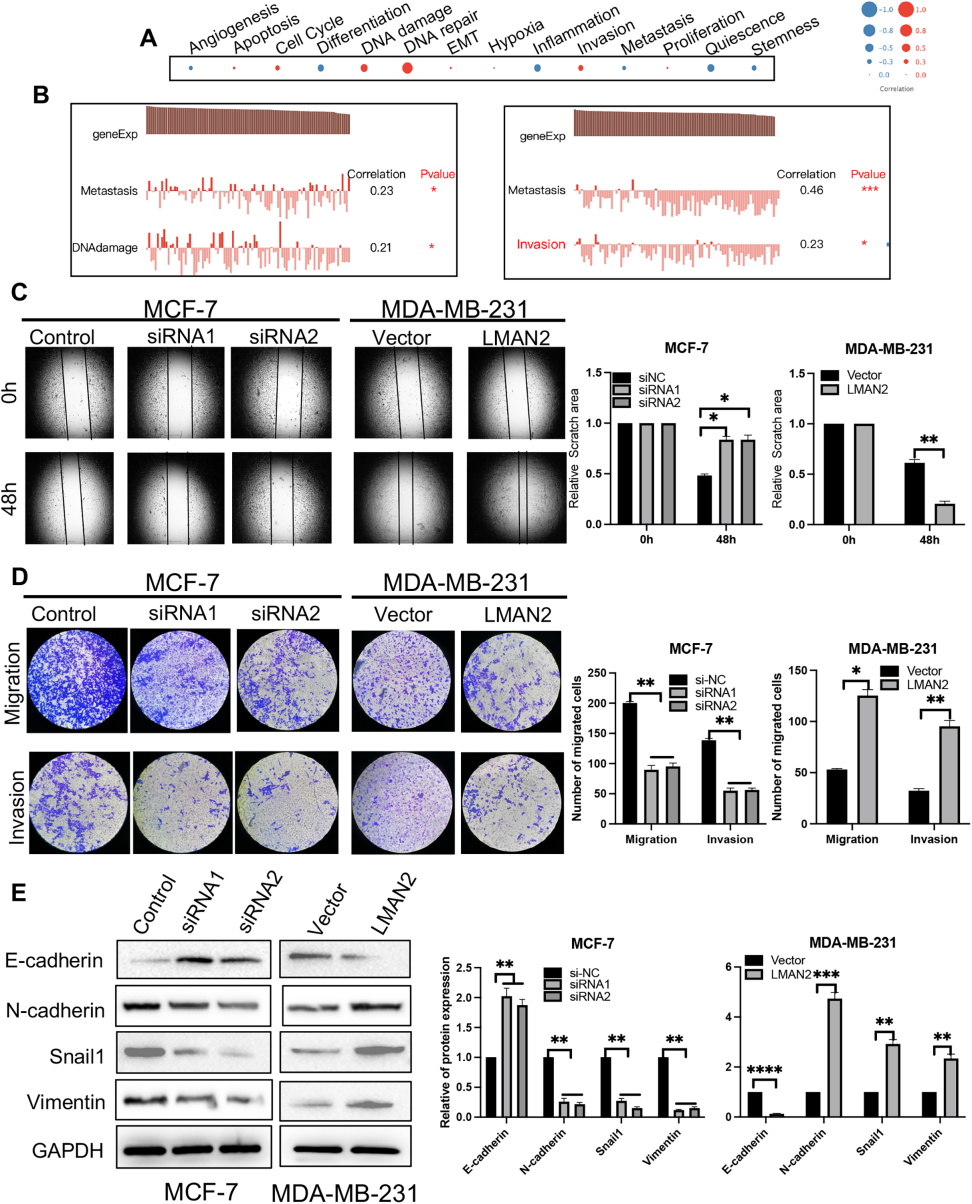
LMAN2 promotes cell mobility. (A) Functional analysis of the correlation between LMAN2 and BC patients using CancerSEA. The bubble size represents the average correlation strength; red indicates a positive correlation, and blue indicates a negative correlation. (B) Detailed functional correlations in the PDX model. (C–D) Results of the wound healing and Transwell chamber assays for assessing migration and invasion abilities. The migratory and invasive cells were stained with Hoechst. (E) Western blot analysis of the expression levels of EMT‐associated proteins (E‐cadherin, N‐cadherin, Snail1, and vimentin). The results of quantitative analysis of cell migration, invasion, and EMT are shown in the right panel. The data are shown as the mean ± SEM of at least three experiments. *p < 0.05, **p < 0.01, ***p < 0.001, ****p < 0.0001.

Intratumoral heterogeneity presents significant obstacles to diagnosing and treating BC. The emergence of single‐cell RNA sequencing technology has afforded great convenience for addressing the functional states of cancer cells at single‐cell resolution. Here, we used the single‐cell database CancerSEA to explore the function of LMAN2 at the single‐cell level. LMAN2 appeared to correlate primarily with the cell cycle, cell invasion, DNA damage, and DNA repair (Figure 4A). Braune EB et al. (Exp0052) showed that high expression of LMAN2 was positively correlated with metastasis and DNA damage (Spearman's coefficients, 0.23 and 0.21, respectively; *p* < 0.05). Similar results were observed in patient‐derived xenografts (CSL‐KO) (Figure 4B). To further elucidate the effect of LMAN2 expression on cell motility, we conducted wound healing and Transwell migration/invasion experiments. A wound scratch assay showed that LMAN2 knockdown hindered MCF‐7 cell migration, whereas its overexpression in MDA‐MB‐231 cells enhanced cell migration (Figure 4C). Consistent results were also evident in the Transwell migration assay: the number of MCF‐7 cells migrating through the interface membrane decreased significantly but increased after LMAN2 overexpression in MDA‐MB‐231 cells (Figure 4D, upper panel). Transwell invasion assays revealed that LMAN2 knockdown considerably reduced the invasive capacity of MCF‐7 cells, while LMAN2 overexpression enhanced the invasion of MDA‐MB‐231 cells (Figure 4D, lower panel). The epithelial–mesenchymal transition (EMT) plays a vital role in metastatic malignancies of epithelial origin. Western blot assays revealed that LMAN2 knockdown suppressed the expression of mesenchymal markers (N‐cadherin, Snail1, and vimentin) and increased the expression of an epithelial marker (E‐cadherin), while LMAN2 overexpression had the opposite effect (Figure 4E).

### 3.4 LMAN2 Promotes Drug Resistance in BC Cells

**FIGURE 5:**
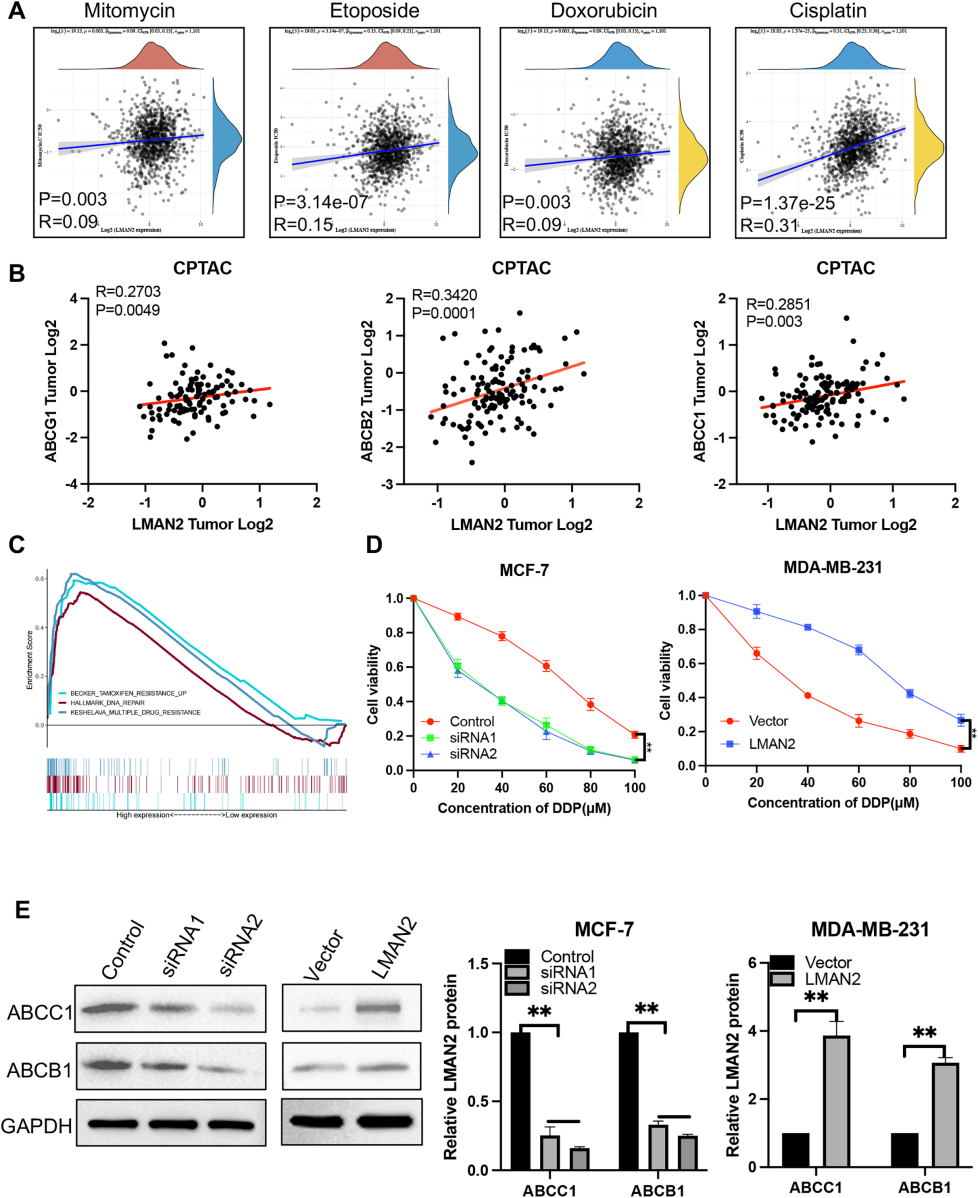
Relationship between drug resistance and LMAN2 expression. (A) Positive correlation analysis of the IC50 score and LMAN2 expression in BC using the GDSC database. (B) Pearson's correlation analysis of LMAN2 expression and multiple drug resistance‐related proteins in BC patients from the CPTAC database. (C) Enrichment plots from GSEA to explore the correlation between LMAN2 expression and multiple drug resistance‐related gene sets based on TCGA‐BRCA datasets. (D) A CCK‐8 assay was performed to evaluate the effect of LAMN2 on cisplatin sensitivity in two BC cell lines. (E) Western blot analysis of the effects of altered LMAN2 expression on MDR1 and MRP1 protein levels. Quantitative analysis of the Western blot bands is shown in the right panel. All values are expressed as the mean ± SEM. **p < 0.01.

Chemoresistance is one of the major obstacles to the successful treatment of human malignancies. We examined the correlation between LMAN2 expression and drug sensitivity using the Genomics of Drug Sensitivity in Cancer (GDSC; https://www.cancerrxgene.org/) database. The findings demonstrated a positive correlation between LMAN2 expression in BC and the IC50 values of several chemotherapeutic drugs, such as mitomycin, etoposide, doxorubicin, and cisplatin (Figure 5A). According to the CPTAC proteomic data, significant positive correlations were identified between LMAN2 and three multidrug‐resistant proteins, namely, ABCG1, ABCB2, and ABCC1 (Figure 5B). GSEA also confirmed that high LMAN2 expression was significantly associated with three gene sets (BECKR_TAMOXIFEN_RESISTANCE_UP, HALLMARK_DNA_REPAIR, and KESHELAVA_MULTIPLE_DRUG_RESISTANCE) (Figure 5C). The CCK‐8 assay illustrated that MCF‐7 cell viability was markedly inhibited by treatment with different concentrations of cisplatin following LMAN2 knockdown. However, LMAN2‐overexpressing MDA‐MB‐231 cells exhibited noticeable resistance to cisplatin (Figure 5D). Western blotting results confirmed that LMAN2 knockdown decreased the expression of MDR1 and MRP1, while LMAN2 overexpression notably promoted their expression (Figure 5E).

### 3.5 LMAN2 Activates the MAPK Pathway in BC Cells

**FIGURE 6:**
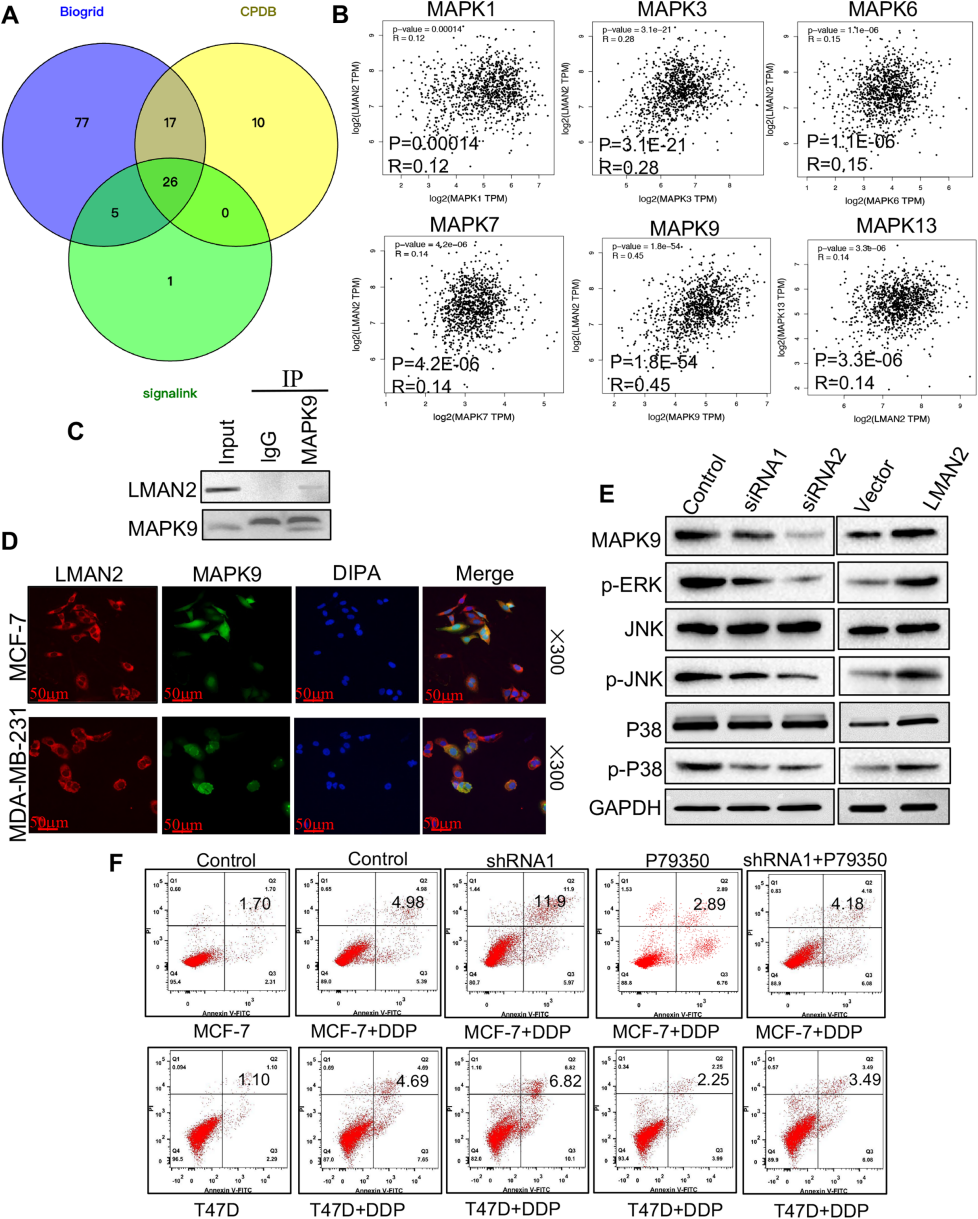
LMAN2 promotes cisplatin resistance in BC by activating the MAPK pathway. (A) Venn diagram showing the number of overlapping genes between the three datasets as indicated. (B) Pearson's correlation analysis between LMAN2 expression and multiple proteins of the MAPK family in BC patients using the GEPIA database. (C) IP assays and Western blotting were conducted to determine the relationship between LMAN2 and MAPK9. (D) Intracellular localization analysis of LMAN2 and MAPK9 by IF assay. The intracellular localization of LMAN2 (red) and MAPK9 (green) is shown. Nuclei (blue) were stained with DAPI. (E) Western blot analysis of the protein levels of MAPK9 and downstream factors of the MAPK signaling pathway. (F) Flow cytometry analysis of the apoptosis of the transfected BC cells in the different treatment groups.

To date, no studies have investigated the potential molecular mechanisms underlying the role of LMAN2 in BC. To investigate the regulatory mechanism of LMAN2, proteins that potentially interact with LMAN2 were screened through the use of three online databases (Biogrid, CPDB, and Signallink). The intersection of these databases revealed 26 prospective proteins (Figure 6A). Among the potential proteins identified, MAPK9 attracted our interest due to confirmation by previous studies of a regulatory link between LMAN2 and the MAPK pathway [11]. In line with our hypothesis, GEPIA revealed a positive correlation between LMAN2 and multiple members of the MAPK family (Figure 6B). Using immunofluorescence (IF), we observed strong colocalization of LMAN2 and MAPK9 in the cytoplasm (Figure 6D). Subsequent IP assays verified a direct interaction between LMAN2 and MAPK9 (Figure 6C). Western blotting was used to quantify alterations in expression of key proteins in the MAPK signaling pathway. LMAN2 knockdown notably inhibited the protein expression of MAPK9, p‐ERK, p‐JNK, and p‐P38. Conversely, LMAN2 upregulation increased the expression of MAPK9 and the formerly phosphorylated proteins (Figure 6E). Reversal experiments revealed that an activator of the MAPK pathway could partially reverse the LMAN2‐induced sensitization of BC cells to cisplatin (Figure 6F). Consequently, LMAN2 potentially regulates chemotherapy resistance in BC via the MAPK signaling pathway.

### 3.6 LMAN2 Knockdown Sensitizes BC Cells to Chemotherapy In Vivo

**FIGURE 7:**
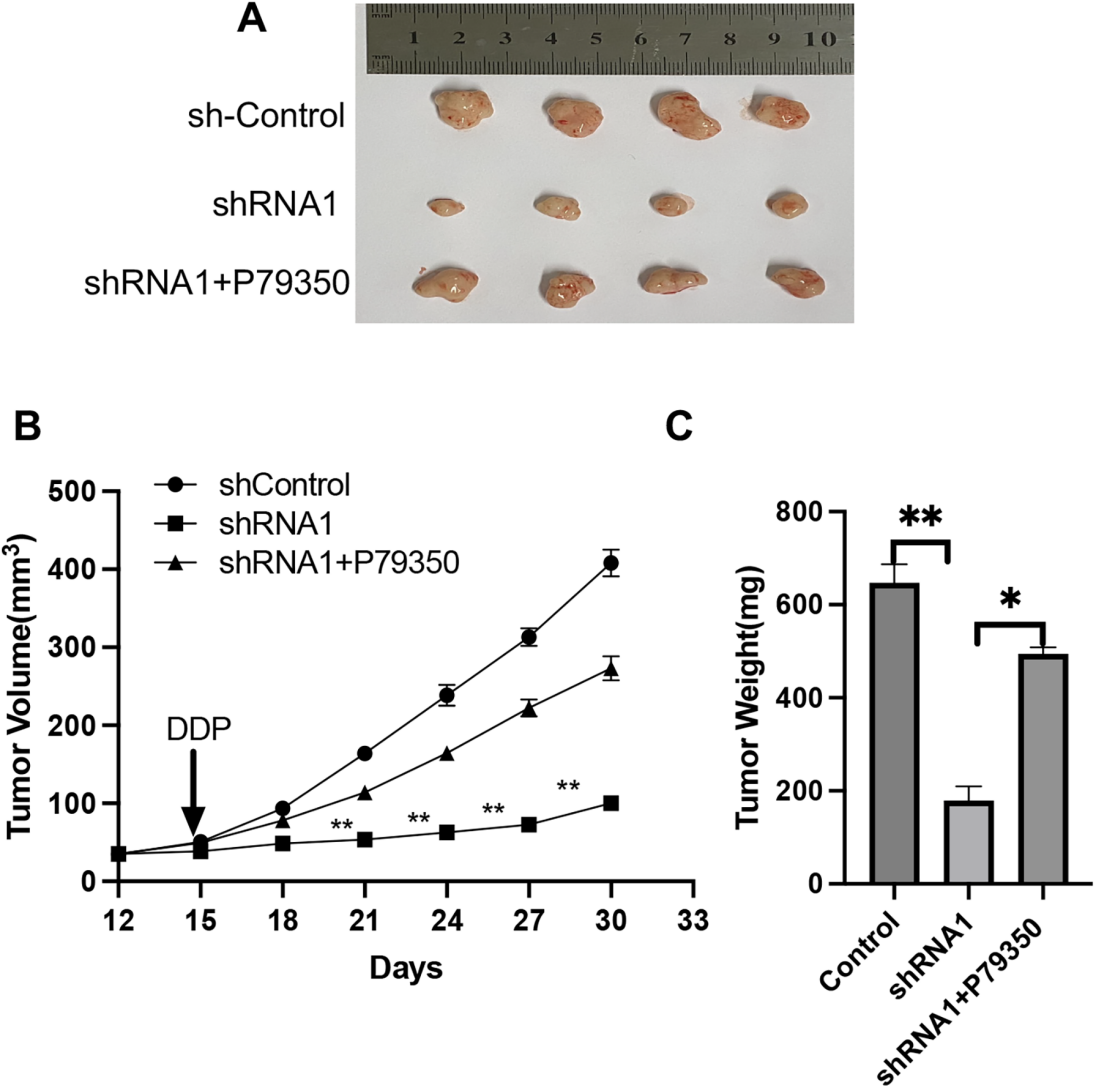
Downregulation of LMAN2 improves the in vivo sensitivity to DDP. (A) Representative images of xenografts in nude mice established via subcutaneous injection of MCF‐7/DDP cells. (B and C) Growth curve and histogram analysis of the volumes and weights of xenograft tumors. *p < 0.05, **p < 0.01.

To investigate whether LMAN2 inhibition enhances the chemosensitivity of BC cells to DDP in vivo, we constructed a BALB/c nude mouse subcutaneous tumor model with DDP‐resistant MCF‐7 cells (MCF‐7/DDP). MCF‐7/DDP cells were transfected with shControl, shLMAN2, or shLMAN2 combined with MAPK activator (P79350). After different treatments, the cells were injected subcutaneously into nude mice, which were subsequently treated with cisplatin. The results suggested that LMAN2 knockdown significantly suppressed tumor growth (Figure 7A) and decreased tumor size (Figure 7B) and tumor weight (Figure 7C) compared with those in the sh‐control group, while the MAPK activator inhibited the sensitivity of MCF‐7/DDP cells to DDP induced by LAMN2 knockdown. Thus, LMAN2 knockdown induced the resensitization of MCF‐7/DDP cells to cisplatin by suppressing the MAPK pathway.

## 4 Discussion

Over the past decade, considerable advancements have been made in the diagnosis and treatment of BC. These achievements have notably increased patient survival rates and have been established as effective therapeutic options for treating breast cancer. Despite surgery, chemotherapy, and radiotherapy being the primary treatment methods for breast cancer, their side effects are considerable [23]. Moreover, targeted therapy has certain drawbacks, including the risk of cardiotoxicity, low rates of effectiveness for single‐drug treatments, inconsistent efficacy, and elevated costs [24]. Moreover, the limited number of clinical studies exploring new drugs, the limited application duration, and the insufficient amount of data related to second‐line and higher‐level treatments have contributed to the ongoing controversy surrounding multiple treatment options.

LMAN2 encodes a type I transmembrane lectin, which acts as a cargo receptor in the transport and sorting of glycoproteins [3, 4]. Previous research has revealed several genes involved in BC that show either upregulated or downregulated expression; these genes are potential diagnostic and prognostic markers for BC [25]. Nevertheless, there are few reports indicating any association between LMAN2 and BC. In this research, we initially used multiple databases to analyze LMAN2 expression in malignant tumors. Multiple databases verified that LMAN2 was significantly upregulated in BC tissues, and elevated LMAN2 expression was associated with clinicopathological characteristics of BC. Patients with elevated LMAN2 expression had a shorter overall survival rate. Furthermore, LMAN2 was implicated in promoting BC cell proliferation, cell cycle progression, and inhibiting apoptosis. In addition, functional experiments revealed that LMAN2 promoted cell migration and invasion and induced EMT in BC cells. In addition, LMAN2 is involved in inducing chemotherapy resistance in BC cells and regulating the expression of multidrug resistance‐related proteins. Interestingly, a majority of these proteins linked to drug resistance are glycoproteins, and LMAN2 principally facilitates the transportation of glycoproteins [26]. Thus, we hypothesized that LMAN2 plays a pivotal role in the transfer of these drug‐resistance‐linked glycoproteins.

To ascertain the mechanism underlying the regulation of chemotherapy resistance by LMAN2, we screened the activation of potential downstream signaling pathways. Through the intersection of the three protein–protein interaction datasets, we concluded that LMAN2 might interact with MAPK9. IP experiments confirmed the direct interaction between LMAN2 and MAPK9, and IF experiments revealed the colocalization of these proteins in the cytoplasm. LMAN2 was also found to be involved in the regulation of several crucial proteins in the MAPK signaling pathway. Accumulating evidence has highlighted that the MAPK pathway plays a crucial role in mediating drug resistance in BC [27]. Activated MAPK cascade kinases (MAPKKK → MAPKK → MAPK) can activate MDR1 transcription [28]. Flow cytometry analysis revealed that LMAN2‐mediated regulation of chemotherapy resistance in BC cells is dependent on the MAPK signaling pathway. In vivo experiments revealed that knocking down LMAN2 expression sensitized transplanted tumors to cisplatin, while concurrent treatment with a MAPK activator reversed this sensitization effect. Thus, we propose that LMAN2 may promote the expression of drug resistance‐related proteins through its impact on the MAPK signaling pathway, thereby influencing chemotherapy resistance in BC.

Although the observations are interesting, this study has several limitations. For instance, a more diverse range of chemotherapeutic drugs should be used to assess the impact of LMAN2 on chemotherapy resistance in BC cell lines, and inhibitory reagents can be used to scrutinize the effects of LMAN2 on the associated signaling pathways. In future studies, proteomics analysis tools such as mass spectrometry could be used to identify protein–protein interactions.

In conclusion, we confirmed that LMAN2 is abundantly expressed in BC tissues and is closely associated with poor prognosis. Furthermore, LMAN2 expression enhances the malignant phenotype and chemotherapy resistance of BC cells. Through mechanistic exploration, we verified that LMAN2 triggers the MAPK signaling pathway by directly binding to MAPK9, thereby contributing to chemotherapy resistance in BC cells. These findings suggest that LMAN2 is a novel diagnostic and prognostic biomarker and a potential therapeutic target for BC patients.

## Author Contributions

**Chen Feng:** data curation (lead), methodology (equal), validation (lead), writing – original draft (lead), writing – review and editing (supporting). **Pingping Li:** conceptualization (equal), formal analysis (equal), project administration (supporting), resources (supporting), software (lead). **Pengtao Liu:** formal analysis (equal), investigation (equal), resources (equal), software (equal), visualization (equal). **Bo Wang:** methodology (equal), project administration (equal), supervision (equal), writing – original draft (equal). **Juan Li:** formal analysis (equal), methodology (equal), software (equal), validation (equal), visualization (equal), writing – original draft (equal). **Peijun Liu:** conceptualization (lead), funding acquisition (lead), writing – original draft (supporting).

## Ethics Statement

All experimental procedures were approved by the Ethics Committee of the First Affiliated Hospital of Xi'an Jiaotong University. Tissues were obtained at the time of surgery from patients after informed consent was written by each patient.

## Conflicts of Interest

The authors declare no conflicts of interest.

## Supporting information


**Data S1.** Biogrid.

**Data S2.** Clinicopathology for Figure 2A.

**Data S3.** CPDB.

**Data S4.** Signallink.

**Figure S1.** Statistical analysis of LMAN2 RNA level after translation with lentivirus in two BC cells.

## Data Availability

All raw data that support the findings of this study are available on request from the corresponding author.
