# Supplementary figures and images for: LMAN2 Promotes Breast Cancer Tumorigenesis and Drug Resistance by Interacting With MAPK9 via Activation of the MAPK Pathway
Source: Cancer Med. 2024 Dec 2;13(23):e70448. doi: 10.1002/cam4.70448 (PMC11609576; doi:10.1002/cam4.70448)

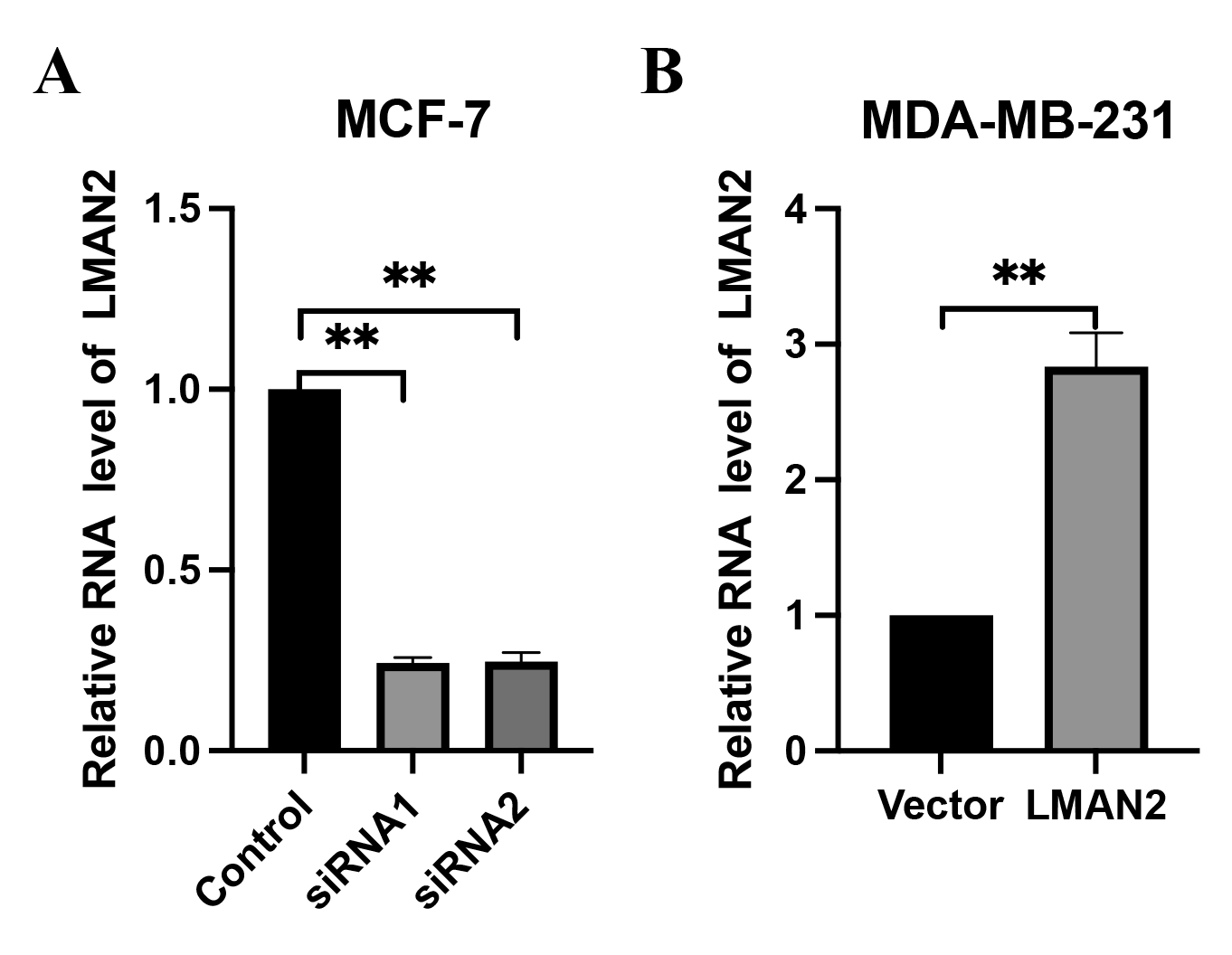

Supplement: Supplementary file 5 — Figure S1. Statistical analysis of LMAN2 RNA level after translation with lentivirus in two BC cells. [file CAM4-13-e70448-s005.tif]
